# Constitutional Thinness and Anorexia Nervosa: A Possible Misdiagnosis?

**DOI:** 10.3389/fendo.2014.00175

**Published:** 2014-10-20

**Authors:** Bruno Estour, Bogdan Galusca, Natacha Germain

**Affiliations:** ^1^Service d’endocrinologie diabète et TCA, Centre Hospitalier Universitaire de Saint-Étienne, Saint Etienne, France

**Keywords:** anorexia nervosa, constitutional thinness, phenotyping, DSM IV, DSM5

## Abstract

Clinical and biological aspects of restrictive anorexia nervosa (R-AN) are well documented. More than 10,000 articles since 1911 and more than 600 in 2013 have addressed R-AN psychiatric, somatic, and biological aspects. Genetic background, ineffectiveness of appetite regulating hormones on refeeding process, bone loss, and place of amenorrhea in the definition are widely discussed and reviewed. Oppositely, constitutional thinness (CT) is an almost unknown entity. Only 32 articles have been published on this topic since 1953. Similar symptoms associating low body mass index, low fat, and bone mass are reported in both CT and R-AN subjects. Conversely, menses are preserved in CT women and almost the entire hormonal profile is normal, except for leptin and PYY. The aim of the present review is to alert the clinician on the confusing clinical presentation of these two situations, a potential source of misdiagnosis, especially since R-AN definition has changed in DSM5.

## Introduction

In a young population of women, between 15 and 30 years old, a low body mass index (BMI) associated with apparent healthy state suggests that the diagnosis of anorexia nervosa (AN). For the same age range, other etiologies of starvation are associated with specific symptoms leading to obvious diagnosis of blood or oncologic pathologies, digestive absorption disorders.

Although restrictive anorexia nervosa (R-AN) definition reported in the “diagnostic and statistical manual of mental disorders” (DSM) is in perpetual evolution, diagnosis remains easy for an experienced clinician. The last issue, DSM5, was published in 2013 (1). Previous DSM IV definition (2) included some psychological elements in the diagnosis such as “refusal to maintain a normal BMI for their age and height, weight loss leading to maintenance of body weight <85% of that expected, fearful of even the slightest weight gain and takes all precautionary measures to avoid weight gain and becoming overweight and disturbance in the way one’s body weight or shape is experienced, undue influence of body weight or shape on self-evaluation, or denial of the seriousness of the current low body weight.” Amenorrhea, i.e., the absence of at least three consecutive menstrual cycles, was the only somatic symptom included in the DSM IV definition. In R-AN women, amenorrhea is related to a blunted/hibernating hypothalamic–pituitary–gonadal (HPG) axis activity. This criterion cannot be applied in pre-menarchal females, females taking oral contraceptives, post-menopausal females, or in case of delayed menarche. Moreover, male gonadal axis status was not mentioned in DSM IV definition. Giving these limitations, a psychiatric taskforce developed a new wide definition meant to be relevant for both genders. Thus, amenorrhea symptom was removed from the current DSM5 definition, entirely based on psychiatric symptoms (1). Major consequences of this truncated definition are further discussed as it can lead to misdiagnosis in front of a thin woman.

## Differences between Anorexia Nervosa and Constitutional Thinness

In a similar population of women, a low BMI associated with apparent healthy state could also suggests that the diagnosis of a not yet well-known entity called constitutional thinness (CT). This natural and physiological low BMI (<18.5 kg/m^2^) state is associated with preserved menses (without contraceptive treatment) and reproductive function leading to constitutional thin families. CT prevalence is unknown in the developed world. These subjects want to gain weight and often consult in this perspective. We recently showed that they failed to gain weight in a controlled overfeeding study (3). Opposite to the presence of amenorrhea that advocates for a diagnosis of R-AN, the absence of amenorrhea advocates for a diagnosis of CT.

Thinness is defined by the World Health Organization which specified three levels of underweight for BMI <18.5 kg/m^2^ (4). Thinness is the only resemblance between R-AN and CT. Indeed, despite the same BMI as AN, CT subjects do not exhibit any typical clinical features such as amenorrhea, fear of weight gain, or hormonal abnormalities commonly seen in R-AN patients.

This review summarizes the main features opposing R-AN and CT including nutritional markers, bone loss physiology, appetite regulating hormones, and how the DSM5 definition can lead to misdiagnoses between those two etiologies of thinness. The results published by our group come from a large cohort of more than 600 R-AN and 100 CT.

**Figure 1:**
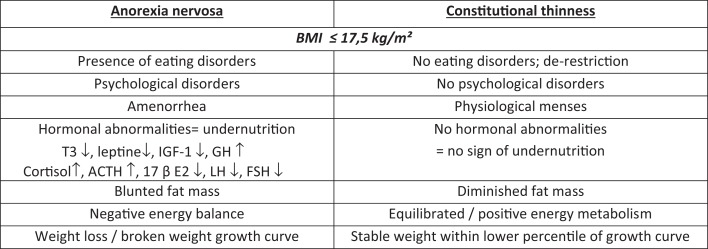
**Psychiatric, hormonal, and energy balance differences between CT and AN**.

Hormonal abnormalities data about R-AN reported so far in literature are commonly accepted and have been showed and published many times by different teams. Most of them are undernutrition markers such as low free-T3 (5, 6), low IGF-1, and elevated GH displaying a GH resistance (7), elevated SHBG (8), and blunted leptin reflecting a decreased fat mass (9). These biological features are adaptive to food restriction and hormonal levels return within normal range after refeeding (10, 11). In 2010, we published biological data on a large R-AN cohort of 200 patients. Despite a correlation between BMI and five specific nutritional or hormonal parameters including leptin, GH, cortisol, free-T3, and IGF-1, we noticed a large inter-individual heterogeneity of these parameters (12). In CT matched for BMI and gender, free-T3, IGF-1, mean 24-h GH levels (sampled six times over the day) are in the normal range (Figure 1). This absence of undernutrition markers is in line with the low levels of leptin, still higher than in AN, and with preserved circadian cycle (13). A girl with low BMI and normal free-T3 or IGF-1 is not a R-AN.

Anorexia nervosa is associated with well-known hypothalamus–pituitary–peripheral axis changes/disturbances such as “pseudo-Cushing syndrome” and hypogonadism. Indeed, the excess of the hypothalamic–pituitary–adrenal (HPA) axis activity includes high CRF (14), increased circadian cortisol levels (15), and rapid escape of cortisol from suppression in response to i.v. dexamethasone (16). Nevertheless, R-AN patients display no clinical Cushing-like features and is considered as a “pseudo-Cushing syndrome.” These abnormalities regress after refeeding (17).

Restrictive anorexia nervosa amenorrhea, the only somatic trait in the DSM IV classification, is the consequence of blunted HPG axis associating low estradiol (18), low free testosterone levels (8), and absence of LH pulsatility (19). This low GnRH activity seems to be dependent on multiple neuropeptides/amino acids influences including opioid (20), gabaergic (21), or serotoninergic activity (22) changes. Refeeding restores menses only in 55–100% of R-AN women (23–25). GnRH pump is the current validated treatment to restore LH pulsatility and to stimulate ovulation (26). This treatment leads to ovulation in 100% of cases, most often between day 10 and 14, and to pregnancy in 55% of cases after 6 stimulations (unpublished data).

Oppositely, normal HPA axis activity suggested by normal six-point (8, 12, 16, 20, 24, 04 h) circadian levels of cortisol was found in CT subjects matched for BMI and gender. Functional HPG axis was also attested by normal LH, FSH, estradiol, and free testosterone plasma level without oral contraception, and preserved fertility (13). A young adult girl with low BMI and normal menses without pills, and normal hormonal profile is not a R-AN.

The alteration of bone quality in AN, a consequence to undernutrition and hormonal abnormalities, is widely accepted. Loss of bone density, correlated with the duration of low BMI (27) and explained by a bone turnover uncoupling (28) leads to increased fractures risk (29), also related with some genetic disturbances (30). An important issue comes from the lack of treatment to increase bone mineral density, except refeeding. Hormonal deficiency supplementation with IGF-1 (31), classical treatment with bisphosphonates (32), or PTH administration (33) are not approved. The role of estradiol in osteoporosis treatment is worldwide accepted, but in R-AN the supplementation is non-effective (34). The association of two hormonal supplementations without side effects such as SDHA and estradiol seems to be effective (35, 36). However, all authors agree on the interest to treat earlier in order to preserve and not to restore bone mineral density.

Bone loss with 44% of osteopenia is also found in 20 years old CT subjects (37). Oppositely to the bone uncoupling seen in R-AN, bone turnover is balanced in CT, with normal bone formation (normal circadian osteocalcin profile) and bone resorption (normal plasma circadian CTX profile). Furthermore, bone microarchitecture measured by pQCT is different (37). Combined normal bone marker and specific microarchitecture profile are other arguments to differentiate CT from R-AN and to avoid a misdiagnosis.

While low food intake characterizes R-AN subjects, equilibrated energy balance was noticed in CT (38). Food restriction behavior of R-AN patients was found to be in opposition with the theoretical action of most of the appetite regulating hormones. Orexigenic gastric hormone ghrelin and the related gene derived peptide obestatin are elevated in all studies without orexigenic effect (13, 39). Ghrelin gene variants (40) or circulating antibodies (41) are proposed to explain the “ghrelin-resistance.” Interestingly, a normal ghrelin level is found in lean anorectic patients (AN) with bingeing and in normal weight bulimic patients. High ghrelin seems to be a mark of restrictive subtype of AN (restrictive only and binging associated) (42). A recently discovered hypothalamic orexigenic peptide called 26 RFa is also increased in R-AN, and oppositely normal in CT (43). These hormones act through the orexigenic neuron and peptide NPY. Conflicting results were reported on pre-prandial plasma NPY (39). A low-circulating NPY could explain the non-efficacy of orexigenic hormones. Recent work showed role of NPY was more complex (44). These results need confirmation by circadian assessment of NPY levels. NPY data are not available in CT yet.

In CT, ghrelin and obestatin are in a normal range (13, 39). Therefore, normal ghrelin levels in a pure R-AN patient are questionable. In this case, misdiagnosis of CT or purging type AN should be discussed (42).

Studies on anorexic appetite regulating hormones as PYY and GLP-1 in AN present with conflicting results (11, 45, 46). PYY level in R-AN was found in normal range in our experience (11), but elevated in another study (47), perhaps due to the misdiagnosis of CT. Indeed, we reported in CT a high-PYY level throughout the day (08, 12, 16, 20, 24, 04 h) (11). CT’s leptin was found lower than in controls but higher than in R-AN with preserved circadian variation. Finally, low level of leptin, another anorexic peptide, found in all R-AN studies (13, 48) is probably more informative on the fat mass than on the eating behavior. Discrepancies found in appetite regulating hormones are in line with differences found in questionnaires measuring restrained eating behavior (DEBQ) or shape concern (EDE). Many questionnaires were proposed for eating disorders phenotyping. EDI questionnaire is a self-report questionnaire used to assess the presence of eating disorders, anorexia nervosa both restricting and binge-eating/purging type (49). EDE questionnaire deals with the frequency in which the patient engages in behaviors indicative of an eating disorder over a 28-day period (restraint, eating, shape, weight concern) (50), DEBQ was developed to measure emotional external and restrain eating (51). All of these psychological scales present pathological scores in AN but not in CT. Moreover, we found an unrestrained eating behavior in CT when compared to controls (3).

For the same, low BMI, anorectic behavior, and adaptive appetite regulating hormone characterizes R-AN patients and distinguishes them from CT patients.

Finally, genetic pathophysiologic role in AN was proposed throughout polymorphism gene studies (52) or familial histories of disease (53). Recently, two studies reported a tendency to the gene hypothesis without the statistical significantly in 421 probants for the first and 1606 probants for the second (54, 55). Significant ratio of chromosome 16p11.2 region duplications was previously noticed in lean patients with autism or schizophrenia (56), without any argument to relate these results to leanness rather than to psychiatric disorders. No genetic signature can be proposed at this moment for the CT patients despite the family pedigree (38). Some studies are in progress but no results are available. Along with all these data on genetic approach, it is important to underline the role of lean subjects’ phenotyping within these large cohorts’ studies.

## Discussion

This review focused on biological differences between R-AN and CT. As CT display specific clinical features and normal hormonal parameters, the misdiagnosis between CT and R-AN is no longer permitted. A very thin girl who claims a normal diet should be heard and not considered as a “liar” AN patient. A biological assessment including leptin, free-T3, and IGF-1 is proposed in order to avoid a misdiagnosis.

This review also focused on conflicting literature data reported in R-AN. Because of the clinical or biological heterogeneity of patients selected for the studies, publications results cannot always be compared and could explained the conflicting data. Therefore, straight and objective classification also called “phenotyping” is required in clinical research in order to obtain better comparable symptoms. For example, the definition shift between DSM IV and DSM5 changes the developing risk of AN, double in a dancer population (57) or in general population (58). Biological assessment including hormonal, nutritional markers, neuroimaging, or questionnaires could help on AN phenotyping. Currently, no biological determination or questionnaire evaluations are required for AN definition in DSM5.

In conclusion, CT represents a well-defined real state of low BMI associating a real weight gain desire, normal nutritional markers except for a mild decreased leptin, a constitutive appetite regulating hormone profile, the presence of menses in young women and low bone mass. While CT diagnosis is still poorly known, the new DSM5 AN definition proposing only psychological traits and no organic symptom is warring. In line with mentioned somatic differences, we advocate complementary biological markers in AN definition in order to avoid the misdiagnosis between AN and CT.

## Conflict of Interest Statement

The authors declare that the research was conducted in the absence of any commercial or financial relationships that could be construed as a potential conflict of interest.

## References

[1] Attia E Becker AE Bryant-Waugh R Hoek HW Kreipe RE Marcus MD Feeding and eating disorders in DSM-5. Am J Psychiatry (2013) 170:1237–9. 10.1176/appi.ajp.2013.13030326 24185238

[2] American Psychiatric Association. Diagnostic and Statistical Manual of Mental Disorders. Washington, DC: American Psychiatric Association (1994). 943 p.

[3] Germain N Galusca B Caron-Dorval D Martin J Pujos-Guillot E Boirie Y Specific appetite, energetic and metabolomics responses to fat overfeeding in resistant-to-bodyweight-gain constitutional thinness. Nutr Diabetes (2014) 4:e126. 10.1038/nutd.2014.17 25027794 PMC5189928

[4] Physical status: the use and interpretation of anthropometry. Report of a WHO Expert Committee. World Health Organ Tech Rep Ser (1995) 854:1–452. 8594834

[5] Chopra IJ Smith SR. Circulating thyroid hormones and thyrotropin in adult patients with protein-calorie malnutrition. J Clin Endocrinol Metab (1975) 40:221–7. 10.1210/jcem-40-2-221 804140

[6] Kiyohara K Tamai H Takaichi Y Nakagawa T Kumagai LF. Decreased thyroidal triiodothyronine secretion in patients with anorexia nervosa: influence of weight recovery. Am J Clin Nutr (1989) 50:767–72. 2508460 10.1093/ajcn/50.4.767

[7] Cabranes JA Almoguera I Santos JL Hidalgo I Borque MM del Olmo J. Somatomedin-C and growth hormone levels in anorexia nervosa in relation to the puberal or post puberal stages. Prog Neuropsychopharmacol Biol Psychiatry (1988) 12:865–71. 10.1016/0278-5846(88)90082-6 3241869

[8] Estour B Pugeat M Lang F Dechaud H Pellet J Rousset H. Sex hormone binding globulin in women with anorexia nervosa. Clin Endocrinol (Oxf) (1986) 24:571–6. 10.1111/j.1365-2265.1986.tb03287.x 3791651

[9] Hebebrand J van der Heyden J Devos R Kopp W Herpertz S Remschmidt H Plasma concentrations of obese protein in anorexia nervosa. Lancet (1995) 346:1624–5. 10.1016/S0140-6736(95)91955-4 7500762

[10] Warren MP. Endocrine manifestations of eating disorders. J Clin Endocrinol Metab (2011) 96:333–43. 10.1210/jc.2009-2304 21159848

[11] Germain N Galusca B Le Roux CW Bossu C Ghatei MA Lang F Constitutional thinness and lean anorexia nervosa display opposite concentrations of peptide YY, glucagon-like peptide 1, ghrelin, and leptin. Am J Clin Nutr (2007) 85:967–71. 17413094 10.1093/ajcn/85.4.967

[12] Estour B Germain N Diconne E Frere D Cottet-Emard JM Carrot G Hormonal profile heterogeneity and short-term physical risk in restrictive anorexia nervosa. J Clin Endocrinol Metab (2010) 95:2203–10. 10.1210/jc.2009-2608 20305007

[13] Tolle V Kadem M Bluet-Pajot MT Frere D Foulon C Bossu C Balance in ghrelin and leptin plasma levels in anorexia nervosa patients and constitutionally thin women. J Clin Endocrinol Metab (2003) 88:109–16. 10.1210/jc.2002-020645 12519838

[14] Hotta M Shibasaki T Masuda A Imaki T Demura H Ling N The responses of plasma adrenocorticotropin and cortisol to corticotropin-releasing hormone (CRH) and cerebrospinal fluid immunoreactive CRH in anorexia nervosa patients. J Clin Endocrinol Metab (1986) 62:319–24. 10.1210/jcem-62-2-319 3001125

[15] Bliss EL Migeon CJ. Endocrinology of anorexia nervosa. J Clin Endocrinol Metab (1957) 17:766–76. 10.1210/jcem-17-6-766 13428843

[16] Estour B Pugeat M Lang F Lejeune H Broutin F Pellet J Rapid escape of cortisol from suppression in response to i.v. dexamethasone in anorexia nervosa. Clin Endocrinol (Oxf) (1990) 33:45–52. 10.1111/j.1365-2265.1990.tb00464.x 2401098

[17] Liu JP Clarke IJ Funder JW Engler D. Studies of the secretion of corticotropin-releasing factor and arginine vasopressin into the hypophysial-portal circulation of the conscious sheep. II. The central noradrenergic and neuropeptide Y pathways cause immediate and prolonged hypothalamic-pituitary-adrenal activation. Potential involvement in the pseudo-Cushing’s syndrome of endogenous depression and anorexia nervosa. J Clin Invest (1994) 93:1439–50. 8163648 10.1172/JCI117121 PMC294157

[18] Baranowska B Zgliczynski S. The role of sex hormones in the mechanism of inhibited LH release in female patients with anorexia nervosa. Acta Endocrinol (Copenh) (1982) 99:334–8. 6803484 10.1530/acta.0.0990334

[19] Allouche J Bennet A Barbe P Plantavid M Caron P Louvet JP. LH pulsatility and in vitro bioactivity in women with anorexia nervosa-related hypothalamic amenorrhea. Acta Endocrinol (Copenh) (1991) 125:614–20. 1789056 10.1530/acta.0.1250614

[20] Valenti S Giusti M Guido R Cavallero D Giordano G. Opioid tonus and luteinizing hormone secretion in anorexia nervosa: priming effect with serotonin precursor L-5-hydroxytryptophan during pulsatile gonadotropin-releasing hormone administration. Biol Psychiatry (1994) 36:609–15. 10.1016/0006-3223(94)90073-6 7833427

[21] Judd SJ Wong J Saloniklis S Maiden M Yeap B Filmer S The effect of alprazolam on serum cortisol and luteinizing hormone pulsatility in normal women and in women with stress-related anovulation. J Clin Endocrinol Metab (1995) 80:818–23. 10.1210/jcem.80.3.7883836 7883836

[22] Lado-Abeal J Rey C Cabezas-Agricola JM Rodriguez A Camarero E Cabezas-Cerrato J. L-hydroxytryptophan amplifies pulsatile secretion of LH in the follicular phase of normal women. Clin Endocrinol (Oxf) (1997) 47:555–63. 10.1046/j.1365-2265.1997.3211126.x 9425395

[23] Hsu LK Crisp AH Harding B. Outcome of anorexia nervosa. Lancet (1979) 1:61–5. 10.1016/S0140-6736(79)90060-6 84126

[24] Viricel J Bossu C Galusca B Kadem M Germain N Nicolau A [Retrospective study of anorexia nervosa: reduced mortality and stable recovery rates]. Presse Med (2005) 34:1505–10. 10.1016/S0755-4982(05)84213-7 16301961

[25] Bergh C Brodin U Lindberg G Södersten P. Randomized controlled trial of a treatment for anorexia and bulimia nervosa. Proc Natl Acad Sci U S A (2002) 99:9486–91. 10.1073/pnas.142284799 12082182 PMC123167

[26] Nillius SJ Fries H Wide L. Successful induction of follicular maturation and ovulation by prolonged treatment with LH-releasing hormone in women with anorexia nervosa. Am J Obstet Gynecol (1975) 122:921–8. 1098466 10.1016/0002-9378(75)90349-x

[27] Rigotti NA Nussbaum SR Herzog DB Neer RM. Osteoporosis in women with anorexia nervosa. N Engl J Med (1984) 311:1601–6. 10.1056/NEJM198412203112503 6504095

[28] Galusca B Bossu C Germain N Kadem M Frere D Lafage-Proust MH Age-related differences in hormonal and nutritional impact on lean anorexia nervosa bone turnover uncoupling. Osteoporos Int (2006) 17:888–96. 10.1007/s00198-005-0063-0 16541206

[29] Faje AT Fazeli PK Miller KK Katzman DK Ebrahimi S Lee H Fracture risk and areal bone mineral density in adolescent females with anorexia nervosa. Int J Eat Disord (2014) 47:458–66. 10.1002/eat.22248 24430890 PMC4053520

[30] Stergioti E Deligeoroglou E Economou E Tsitsika A Dimopoulos KD Daponte A Gene receptor polymorphism as a risk factor for BMD deterioration in adolescent girls with anorexia nervosa. Gynecol Endocrinol (2013) 29:716–9. 10.3109/09513590.2013.798275 23772785

[31] Grinspoon S Baum H Lee K Anderson E Herzog D Klibanski A. Effects of short-term recombinant human insulin-like growth factor I administration on bone turnover in osteopenic women with anorexia nervosa. J Clin Endocrinol Metab (1996) 81:3864–70. 10.1210/jcem.81.11.8923830 8923830

[32] Miller KK Meenaghan E Lawson EA Misra M Gleysteen S Schoenfeld D Effects of risedronate and low-dose transdermal testosterone on bone mineral density in women with anorexia nervosa: a randomized, placebo-controlled study. J Clin Endocrinol Metab (2011) 96:2081–8. 10.1210/jc.2011-0380 21525157 PMC3135194

[33] Fazeli PK Wang IS Miller KK Herzog DB Misra M Lee H Teriparatide increases bone formation and bone mineral density in adult women with anorexia nervosa. J Clin Endocrinol Metab (2014). 10.1210/jc.2013-4105 24456286 PMC3973785

[34] Misra M Klibanski A. Bone health in anorexia nervosa. Curr Opin Endocrinol Diabetes Obes (2011) 18:376–82. 10.1097/MED.0b013e32834b4bdc 21897220 PMC3679194

[35] Divasta AD Feldman HA Giancaterino C Rosen CJ Leboff MS Gordon CM. The effect of gonadal and adrenal steroid therapy on skeletal health in adolescents and young women with anorexia nervosa. Metabolism (2012) 61:1010–20. 10.1016/j.metabol.2011.11.016 22257645 PMC3465078

[36] DiVasta AD Feldman HA Beck TJ LeBoff MS Gordon CM. Does hormone replacement normalize bone geometry in adolescents with anorexia nervosa? J Bone Miner Res (2014) 29:151–7. 10.1002/jbmr.2005 23744513 PMC3812374

[37] Galusca B Zouch M Germain N Bossu C Frere D Lang F Constitutional thinness: unusual human phenotype of low bone quality. J Clin Endocrinol Metab (2008) 93:110–7. 10.1210/jc.2007-1591 17956951

[38] Bossu C Galusca B Normand S Germain N Collet P Frere D Energy expenditure adjusted for body composition differentiates constitutional thinness from both normal subjects and anorexia nervosa. Am J Physiol Endocrinol Metab (2007) 292:E132–7. 10.1152/ajpendo.00241.2006 16912058

[39] Germain N Galusca B Grouselle D Frere D Tolle V Zizzari P Ghrelin/obestatin ratio in two populations with low bodyweight: constitutional thinness and anorexia nervosa. Psychoneuroendocrinology (2009) 34:413–9. 10.1016/j.psyneuen.2008.10.001 18995969

[40] Ando T. Ghrelin gene variants and eating disorders. Vitam Horm (2013) 92:107–23. 10.1016/B978-0-12-410473-0.00004-0 23601422

[41] Terashi M Asakawa A Harada T Ushikai M Coquerel Q Sinno MH Ghrelin reactive autoantibodies in restrictive anorexia nervosa. Nutrition (2011) 27:407–13. 10.1016/j.nut.2011.01.002 21392704

[42] Germain N Galusca B Grouselle D Frere D Billard S Epelbaum J Ghrelin and obestatin circadian levels differentiate bingeing-purging from restrictive anorexia nervosa. J Clin Endocrinol Metab (2010) 95:3057–62. 10.1210/jc.2009-2196 20339027

[43] Galusca B Jeandel L Germain N Alexandre D Leprince J Anouar Y Orexigenic neuropeptide 26RFa: new evidence for an adaptive profile of appetite regulation in anorexia nervosa. J Clin Endocrinol Metab (2012) 97:2012–8. 10.1210/jc.2011-3396 22466335

[44] Södersten P Nergårdh R Bergh C Zandian M Scheurink A. Behavioral neuroendocrinology and treatment of anorexia nervosa. Front Neuroendocrinol (2008) 29:445–62. 10.1016/j.yfrne.2008.06.001 18602416

[45] Sedlackova D Kopeckova J Papezova H Hainer V Kvasnickova H Hill M Comparison of a high-carbohydrate and high-protein breakfast effect on plasma ghrelin, obestatin, NPY and PYY levels in women with anorexia and bulimia nervosa. Nutr Metab (Lond) (2012) 9:52. 10.1186/1743-7075-9-52 22681985 PMC3533897

[46] Lawson EA Eddy KT Donoho D Misra M Miller KK Meenaghan E Appetite-regulating hormones cortisol and peptide YY are associated with disordered eating psychopathology, independent of body mass index. Eur J Endocrinol (2011) 164:253–61. 10.1530/EJE-10-0523 21098684 PMC3677777

[47] Utz AL Lawson EA Misra M Mickley D Gleysteen S Herzog DB Peptide YY (PYY) levels and bone mineral density (BMD) in women with anorexia nervosa. Bone (2008) 43:135–9. 10.1016/j.bone.2008.03.007 18486583 PMC2493518

[48] Grinspoon S Gulick T Askari H Landt M Lee K Anderson E Serum leptin levels in women with anorexia nervosa. J Clin Endocrinol Metab (1996) 81:3861–3. 10.1210/jc.81.11.3861 8923829

[49] Garner DM Olmstead MP Polivy J. Development and validation of a multidimensional eating disorder inventory for anorexia nervosa and bulimia. Int J Eat Disord (1983) 2:15–34. 10.1002/1098-108X(198321)2:2<15::AID-EAT2260020203>3.0.CO;2-617147952

[50] Cooper Z Fairburn CG. The eating disorders examination: a semistructured interview for the assessment of the specific psychopathology of eating disorders. Int J Eat Disord (1987) 6:1–8. 10.1002/1098-108X(198701)6:1<1::AID-EAT2260060102>3.0.CO;2-911054796

[51] Van Strien T Friejters JE Bergers GPA Defares PB. The Dutch eating behavior questionnaire for assessment of restrained, emotional and external eating behavior. Int J Eat Disord (1986) 5:295–315. 10.1002/1098-108X(198602)5:2<295::AID-EAT2260050209>3.0.CO;2-T

[52] Enoch MA Kaye WH Rotondo A Greenberg BD Murphy DL Goldman D. 5-HT2A promoter polymorphism -1438G/A, anorexia nervosa, and obsessive-compulsive disorder. Lancet (1998) 351:1785–6. 10.1016/S0140-6736(05)78745-6 9635956

[53] Woodside DB Field LL Garfinkel PE Heinmaa M. Specificity of eating disorders diagnoses in families of probands with anorexia nervosa and bulimia nervosa. Compr Psychiatry (1998) 39:261–4. 10.1016/S0010-440X(98)90033-5 9777277

[54] Pinheiro AP Bulik CM Thornton LM Sullivan PF Root TL Bloss CS Association study of 182 candidate genes in anorexia nervosa. Am J Med Genet B Neuropsychiatr Genet (2010) 153B:1070–80. 10.1002/ajmg.b.31082 20468064 PMC2963154

[55] Boraska V Franklin CS Floyd JA Thornton LM Huckins LM Southam L A genome-wide association study of anorexia nervosa. Mol Psychiatry (2014). 10.1038/mp.2013.187 24514567 PMC4325090

[56] Jacquemont S Reymond A Zufferey F Harewood L Walters RG Kutalik Z Mirror extreme BMI phenotypes associated with gene dosage at the chromosome 16p11.2 locus. Nature (2011) 478:97–102. 10.1038/nature10406 21881559 PMC3637175

[57] Arcelus J Witcomb GL Mitchell A. Prevalence of eating disorders amongst dancers: a systemic review and meta-analysis. Eur Eat Disord Rev (2013) 22(2):92–101. 10.1002/erv.2271 24277724

[58] Brown TA Holland LA Keel PK. Comparing operational definitions of DSM-5 anorexia nervosa for research contexts. Int J Eat Disord (2014) 47:76–84. 10.1002/eat.22184 24013875

